# Correlation between folic acid and hemoglobin in pregnant women: cross-sectional study from 2017 to 2019

**DOI:** 10.1590/0034-7167-2024-0568

**Published:** 2026-03-16

**Authors:** Tazla Ingride de Souza Lins, Geyson Alves Marinho, Vitória Caroline Santana Chaves da Silva, Myrella Maria de Sena, Suzana Lins da Silva, Malaquias Batista, Maria de Fátima Costa Caminha

**Affiliations:** I Instituto de Medicina Integral Prof. Fernando Figueira. Recife, Pernambuco, Brazil; II Faculdade Pernambucana de Saúde. Recife, Pernambuco, Brazil

**Keywords:** Anemia, Folic Acid, Folic Acid Deficiency, Pregnancy, Hemoglobin., Anemia, Ácido Fólico, Deficiencia de Ácido Fólico, Embarazo, Hemoglobina.

## Abstract

**Objectives:**

to correlate folic acid levels with hemoglobin levels in pregnant women.

**Methods:**

a cross-sectional study conducted between 2017 and 2019 with 950 pregnant women based on the cohort database “Nutrition and Infection: The Problem Revisited Due to the Microcephaly Outbreak”. Data were analyzed using Stata 12.1. Spearman’s correlation and Pearson’s chi-square tests were applied, with a significance level of 5%.

**Results:**

folic acid and hemoglobin levels were adequate (>5.38 ng/ml) in 90.9% of participants. Hemoglobin concentration was adequate (≥ 11 g/dL) in 80.8% of pregnant women. Pregnant women with hemoglobin ≤ 11 g/dL had 13.2% of inadequate folic acid, while in the group with hemoglobin ≥ 11 g/dL, 8.1% had inadequate folic acid (p=0.031).

**Conclusions:**

an association was observed between hemoglobin and folic acid levels in the pregnant women assessed, reinforcing the importance of nutritional monitoring during prenatal care.

## RESUMEN

**Objetivos:**

correlacionar los niveles de ácido fólico con los de hemoglobina en embarazadas.

**Métodos:**

estudio transversal realizado entre 2017 y 2019 con 950 embarazadas, basado en la base de datos de cohorte “Nutrición e Infección: El Problema Revisado Debido al Brote de Microcefalia”. Los datos se analizaron con Stata 12.1. Se aplicaron las pruebas de correlación de Spearman y chi-cuadrado de Pearson, con un nivel de significancia del 5%.

**Resultados:**

los niveles de ácido fólico y hemoglobina fueron adecuados (>5,38 ng/ml) en el 90,9% de las participantes. La concentración de hemoglobina fue adecuada (≥ 11 g/dL) en el 80,8% de las gestantes. Las gestantes con hemoglobina ≤ 11 g/dL presentaron un 13,2% de deficiencia de ácido fólico, mientras que en el grupo con hemoglobina ≥ 11 g/dL, el 8,1% presentó deficiencia de ácido fólico (p=0,031).

**Conclusiones:**

se observó una asociación entre los niveles de hemoglobina y ácido fólico en las gestantes evaluadas, lo que refuerza la importancia del seguimiento nutricional durante la atención prenatal.

## INTRODUCTION

Pregnancy is a period characterized by significant physiological changes that require essential nutrients for proper fetal development. Among these nutrients, folic acid plays a crucial role, with demand that can increase ten to 20 times compared to the non-pregnant period^(1)^.

Folic acid is the synthetic form of folate, discovered by hematologist Lucy Wills in 1931 for macrocytic anemia treatment in pregnant women^(2)^. This nutrient is essential for cell division, tissue growth, proliferation and growth of neuronal cells, and the synthesis of neurotransmitters^(2-4)^. Folic acid is also essential in preventing neural tube birth defects, which can be reduced by supplementing this micronutrient early in pregnancy^(5)^.

Folic acid deficiency remains a problem in many countries around the world, especially in low-income regions. Fortifying foods, such as wheat and corn flours, with iron and folic acid has been an effective strategy to combat this deficiency, reaching pregnant women with low incomes and unplanned pregnancies^(6-11)^. Since 1998, it has been recommended to consume 400 mcg/day of fortified foods or supplements, in addition to folic acid obtained through diet^(12)^.

In addition to folic acid requirements during pregnancy, other nutrients such as vitamin B12 and iron are essential. Deficiencies can cause certain types of anemia^(13)^. The epidemiology and etiology of anemia are multifactorial and involve a complex interaction of causes^(14-16)^. Large-scale iron and folic acid supplementation and dietary fortification may improve hemoglobin/anemia status^(17-19)^.

The World Health Organization recommends iron and folic acid supplementation as part of prenatal care to reduce the risks to which mother and child are exposed^(20)^. Pregnant women who do not take iron and folic acid supplements have a three times greater risk of developing anemia^(21)^.

The interaction with iron deficiency anemia is likely to have a significant confounding effect on assessing the contribution of folic acid deficiency to anemia. Iron deficiency causes red blood cell changes that are opposite to those seen in folic acid deficiencies, and concurrent iron deficiency may mask folic acid deficiency^(22,23)^.

There is evidence that hemoglobin concentration is inversely associated with erythrocyte folic acid concentrations; however, this association was not found in pregnant women^(24)^. Thus, the study aimed to correlate folic acid levels with hemoglobin levels in pregnant women treated at a maternal and child referral hospital in northeastern Brazil.

## OBJECTIVES

To analyze the correlation of folic acid levels with hemoglobin levels during pregnancy.

## METHODS

### Ethical aspects

The study adhered to the ethical guidelines established by Resolution No. 510/2016 of the Brazilian National Health Council and was approved by the Human Research Ethics Committee of the Instituto de Medicina Integral Prof. Fernando Figueira (IMIP) under CAAE 71259323.8.0000.5201, approval number 6.256.348, issued on August 23, 2023. All participants provided written informed consent prior to enrollment.

### Study design, period and location

This is a cross-sectional study with a single-point design, using data from a larger cohort study conducted between 2017 and 2019 entitled “Nutrition and Infection: The Problem Revisited Due to the Microcephaly Outbreak”. The study was conducted at the IMIP Women’s Care Center, specifically in the prenatal outpatient clinic. The manuscript was written in accordance with STrengthening the Reporting of OBservational studies in Epidemiology recommendations for observational studies, ensuring clarity and replicability of the methodology used.

Data collection for the cohort began in April 2017 and was completed in March 2019 by the Integrated Nutrition and Health Study Group at IMIP. The period considered for this study, from 2017 to 2019, corresponds to a total of 950 pregnant women included in the database with available folic acid and hemoglobin results.

### Study sampling and inclusion and exclusion criteria

The original study sample was a convenience sample of 1,469 pregnant women. Women were approached while awaiting their prenatal appointment. The research objectives and procedures were detailed, and pregnant women decided whether to comply with the research protocol, signed the Informed Consent Form, and answered the questions on the form. Anthropometric measurements (weight and height) were taken and blood samples were collected for laboratory tests, including a folic acid test. Hemoglobin test results were retrieved from women’s medical records.

The population/sample of the current study consisted of pregnant women from the original research database with results for folic acid and hemoglobin. Pregnant women who did not have results for folic acid and hemoglobin were excluded.

Pregnant women’s sociodemographic variables (age, race, years of education, origin, occupation, and *per capita* income), obstetric variables (number of pregnancies, history of miscarriage, trimester of prenatal care initiation), clinical variables (serum folic acid and hemoglobin levels), and nutritional variables (Atalah classification) were used. The Atalah classification was based on the pre-gestational Body Mass Index (BMI), considering the cut-off points established for each gestational age, according to the method proposed by Atalah.

Folic acid levels were measured at the time pregnant women were enrolled in the study, regardless of their trimester of pregnancy. Hemoglobin levels were obtained from the results available in pregnant women’s medical records, considering the first hemoglobin level recorded during the pregnancy, corresponding to the time pregnant women entered the study.

### Study protocol

An ad hoc database was created with information of interest to meet the objectives of the current study.

### Analysis of results and statistics

Statistical analysis was performed in Stata 12.1 using Spearman’s correlation test and Pearson’s chi-square test. For statistical purposes, a p-value < 5% was considered.

## RESULTS

Table 1 describes the frequency of pregnant women’s sociodemographic characteristics. According to the data, most pregnant women were between 20 and 35 years old, brown, with 12 or more years of education, and living in urban areas. Furthermore, most were in a stable relationship or married. However, despite this profile, more than half of participants were not in the labor market at the time of data collection, and approximately 40% had a *per capita* income of less than half the minimum wage.

**Table 1:** Frequency distribution of sociodemographic characteristics of pregnant women treated at a maternal and child referral hospital in northeastern Brazil (April 2017 to July 2018)

Variables	n^*^(%)
**Age (N = 950)**	
<= 19 years	100 (10.5)
20 to 35 years	708 (74.5)
36 to 47 years	142 (14.9)
**Race (N = 944)**	
White	190 (20.1)
Black	164 (17.4)
Brown	507 (53.7)
Indigenous	17 (1.8)
Yellow	66 (7.0)
**Years of study (N = 949)**	
Up to 8	117 (12.3)
9 to 11	117 (12.3)
12 or older	715 (75.3)
**Origin (N = 950)**	
Urban	921 (96.9)
Rural	29 (3.1)
**Marital status (N = 950)**	
Single/separated/widowed	193 (20.3)
Married/common-law relationship	757 (79.7)
**Paid occupation (N = 948)**	
Yes	459 (48.4)
No	489 (51.6)
**Per capita income (MW) (N = 867)**	
<1/2 MWs	361 (41.6)
1/2 to 1 MWs	296 (34.1)
>1 MW	210 (24.2)

MW - minimum wage;

* The sample varied according to the information collected.

Table 2 presents the gestational characteristics, anthropometric data, lifestyle habits, and hemoglobin and folic acid levels. Most of pregnant women were primigravidae and were overweight or obese according to the Atalah classification.

**Table 2:** Frequency distribution of obstetric, nutritional, and clinical characteristics of pregnant women treated at a maternal and child referral hospital in northeastern Brazil (April 2017 to July 2018)

Variables	n^*^(%)
**Pregnancy (N = 950)**	
First pregnancy	367 (38.6)
Second pregnancy	280 (29.5)
Multigravida	303 (31.9)
**History of miscarriage (N = 585)**	
Yes	243 (41.5)
No	342 (58.5)
**Trimester of early prenatal care (N = 891)**	
1st trimester	620 (69.6)
2nd trimester	271 (30.4)
**Atalah classification (N = 942)**	
Underweight	119 (12.6)
Adequate	313 (33.2)
Overweight	273 (29.0)
Obesity	237 (25.2)
**GA in folic acid collection (N = 950)**	
1st trimester	313 (32.9)
2nd trimester	632 (66.5)
3rd trimester	5 (0.5)
**Use of folic acid supplement (N = 950)**	
Yes	630 (66.3)
No	320 (33.7)
**Hemoglobin (N = 950)**	
<11 g/dl	182 (19.2)
>=11 g/dl	768 (80.8)
**Folic acid dosage (N = 950)**	
≤ 5.38 ng/ml	86 (9.1%)
> 5.38 ng/ml	864 (90.9%)

GA - gestational age;

* The sample varied according to the information collected.

Approximately 70% began prenatal care in the first trimester, indicating relatively early access to healthcare. It was also observed that over 40% of participants reported a previous history of miscarriage. Regarding the timing of folic acid collection, most measurements were performed in the second trimester of pregnancy, reflecting the dynamics of participant inclusion in the study. Concerning supplementation, approximately two-thirds of pregnant women reported using folic acid during pregnancy (Table 2).

Folic acid dosage in pregnant women was adequate (> 5.38 ng/ml) in 90.9% of participants. Hemoglobin concentration was adequate (≥ 11 g/dL) in 80.8% of pregnant women.

The correlation between folic acid and hemoglobin values showed a Spearman’s correlation coefficient of 0.08, which translates into a weak correlation.

Table 3 shows the association between hemoglobin and folic acid levels in pregnant women. In the group with hemoglobin ≤11 g/dL, 13.2% of pregnant women had folic acid deficiency. Furthermore, in the group of pregnant women with hemoglobin ≥11 g/dL, only 8.1% had folic acid deficiency. This result was statistically significant (p-value = 0.031).

**Table 3:** Association between hemoglobin levels and folic acid deficiency in pregnant women treated at a maternal and child referral hospital for northeastern Brazil (April 2017 to July 2018)

Hemoglobin	Folic acid deficiency		*p* value^*^
	Yes	No	
			0.031
<11 g/Dl	24 (13.2%)	158 (86.8%)	
≥11 g/Dl	62 (8.1%)	706 (91.9%)	

* *Chi-square test.*

## DISCUSSION

Adequate nutrient intake during pregnancy is recognized as important by the scientific community^(25)^. Considering the relevance of folic acid and hemoglobin adequacy for maternal health, the current study found adequate folic acid and hemoglobin levels in most pregnant women.

It was observed that the majority of participants presented sociodemographic characteristics that indicate greater access to healthcare services, such as high levels of education and early initiation of prenatal care.

When compared with the literature, research carried out with 1,107 pregnant women revealed that 42.4% had folic acid insufficiency, despite free access to folic acid supplements^(26)^. In another study, which included 250 pregnant women, folic acid deficiency was found in 22.4%. Of these, 4.4% used folic acid supplements, while in the present study, 66.3% of pregnant women reported having used folic acid supplements^(27)^. Another study, published in 2023, assessed the prevalence of anemia by hemoglobin levels in pregnant women, including 208 participants, and found that 42.3% had anemia^(21)^.

Studies have shown that non-folic acid supplementation is a major factor associated with folic acid deficiency or insufficiency and anemia during pregnancy. Due to low supplement adherence, food fortification appears to be a truly important complementary micronutrient intervention^(28,29)^.

In an attempt to assess the expected benefits of food fortification policy in Brazil, a cross-sectional study assessed the prevalence of anemia during pregnancy before and after flour fortification with iron and folic acid, investigating 12,119 pregnant women using the public health system in 13 municipalities across Brazil’s five regions. The analysis revealed no significant increase in hemoglobin levels after fortification (p = 0.325)^(30)^.

Another recent study, conducted in Ireland and involving 759 pregnant women followed through the ROLO longitudinal cohort, assessed dietary intake of iron, folate, and vitamin B12 during pregnancy and their correlation with maternal hemoglobin levels and fetal growth. Despite inadequate iron and folate intake observed in all trimesters, hemoglobin levels remained adequate at 13 and 28 weeks of gestation^(31)^.

These data, however, are conflicting, as a study conducted in Teresina, Piauí, adopted a longitudinal approach to investigate the relationship between anemia and flour fortification containing iron and folic acid in pregnant women. The results of this study demonstrated a significant change in mean hemoglobin levels after the implementation of fortification. Before the intervention, mean hemoglobin levels were 11.7 g/dL, increasing substantially to 12.4 g/dL after fortification. Furthermore, the prevalence of anemia decreased considerably from 27.2% in the unfortified group to 11.5% in the fortified group^(32)^.

In another study conducted in Maringá, Paraná, the effects of flour fortification on the prevalence of anemia in pregnant women were investigated. The medical records of 366 pregnant women before and 419 pregnant women after mandatory flour fortification were analyzed. The results showed a low prevalence of anemia before (12.3%) and after (9.4%) fortification, with no statistically significant differences. However, the post-fortification group had a mean hemoglobin concentration 0.17 g/dL higher than the unfortified group^(19)^.

In this study, it was observed that some pregnant women did not use folic acid supplementation during pregnancy. No specific investigation into the reasons for this phenomenon was conducted. However, it is possible that factors such as late initiation of prenatal care, lack of a medical prescription, and refusal by the pregnant woman herself may have contributed to this situation.

Studies suggest that pregnant women who live with a partner, have a higher level of education, are first-time mothers, and who began prenatal care in the first trimester have a lower prevalence of folic acid deficiency and anemia^(33-35)^, which supports the data from the pregnant women in the present study and may have contributed to the low levels of folic acid deficiency and hemoglobin among pregnant women. It is also noteworthy that the study was conducted in a referral hospital, and the majority (70.1%) of pregnant women began prenatal care in the first trimester and, consequently, iron and folic acid supplementation.

A study published in 2021, which assessed the prevalence of consumption of iron and folic acid supplements and associated factors among pregnant women, including 330 pregnant women, found that only 10.3% used them correctly during the first trimester and the main factor associated with low supplement consumption was the late start of prenatal care^(35)^.

A lack of folic acid impairs the ability of red blood cells to remain intact in the face of oxidizing agents^(36)^. Megaloblastic macrocytic anemias most often result from vitamin B12 and folic acid deficiencies. Studies show that prior to mandatory national folic acid fortification programs and guidance on periconceptional supplementation of this micronutrient, folic acid deficiency was the second most common cause of anemia during pregnancy^(37,38)^.

A randomized clinical trial demonstrated that combined interventions of nutritional education and iron-folic acid supplementation resulted in an increase of ~10g/L in hemoglobin and a 69% reduction in anemia, followed by a significant decrease in neonatal mortality^(39)^.

Another systematic review with meta-analysis published in 2021, with a total of 439,649 women in the included studies, which assessed the effects of vitamin and mineral supplementation during pregnancy on maternal, birth, child health, and developmental outcomes, found that iron supplementation along with folic acid showed a significant reduction (48%) in the risk of maternal anemia^(40)^.

Meanwhile, another study, also published in 2021, assessed whether the inclusion of folic acid in weekly iron supplements conferred any benefit on hemoglobin concentration or reduction of anemia, finding that, after 16 weeks of including folic acid in the supplementation, there was no difference in hemoglobin concentrations^(41)^.

It is worth noting that, due to the lack of folic acid fortification in Malaysia, where the aforementioned study was conducted, the female population in this region likely has one of the lowest folic acid levels globally, as evidenced by folic acid deficiency in 84% of women, a much higher percentage compared to the present study. It should also be noted that the study duration was only 16 weeks. Populations with higher baseline folic acid status and mandatory fortification may have a better response in hemoglobin levels^(42)^.

In relation to the factors associated with the risk of anemia, a study that aimed to identify sociodemographic characteristics related to anemia during pregnancy observed that women were more likely to have anemia during the periconceptional period when they were younger, multiparous, single, had a low level of education, low family income, were unemployed during pregnancy, and lived in rural areas or areas with difficult access to healthcare services^(43)^.

The low folic acid and hemoglobin deficiency identified in this study may be attributed to the fact that most pregnant women in the study were over 19 years old, married or in a stable relationship, and had 12 or more years of education, residing in urban areas. These characteristics suggest a potential association between higher educational levels, marital stability, and a greater likelihood of adopting appropriate prenatal health practices, such as early initiation of prenatal care and greater adherence to micronutrient supplementation.

Furthermore, studies show that maternal BMI is associated with the risk of anemia^(44-46)^. In Brazil, pregnant women with adequate or overweight weight had significantly lower chances of being anemic, compared to women with low weight (adequate versus low weight (0.79Odds Ratio; 95% Confidence Interval; 0.66 to 0.94); overweight versus low weight (0.42Odds Ratio; 95% Confidence Interval; 0.42 to 0.66). Supporting the data from the pregnant women in the study, the majority had an adequate BMI (32.9%) and were overweight (28.6%)^(30)^.

In Brazil, food fortification guidelines, since it became mandatory, have been based on iron and folic acid^(31)^. Recent studies reinforce the relevance of supplementation strategies to reduce anemia in pregnant women, including evidence-based interventions for lowand middle-income populations^(21,47)^.

Supplementation with this vitamin and mineral is routine early in prenatal care, which may have resulted in the significant correlation found between hemoglobin and folic acid levels in this study. Since this was a cross-sectional study, it is not possible to establish causal relationships between folic acid and hemoglobin levels. The findings, however, reinforce the importance of nutritional monitoring during pregnancy.

### Study limitations

This study has some limitations. The main one is that it obtained results from a sample of women who gave birth at a maternal and child referral hospital, the majority of whom began prenatal care in the first trimester.

Furthermore, it is noteworthy that although most participants began prenatal care early, folic acid levels were mostly measured in the second trimester of pregnancy, which may have influenced the representativeness of the observed serum levels. This characteristic may have contributed to the low prevalence of folic acid deficiency and the adequate hemoglobin levels found in the sample.

Furthermore, as this was a secondary database analysis, it was not possible to include other variables potentially related to folic acid deficiency, such as pregnancy planning and use of supplementation in the preconception period.

Finally, it is important to emphasize that the present study used only bivariate analyses, without adjustment for potential confounding factors, due to the limitations inherent in the available database. This methodological limitation should be considered when interpreting the results, especially regarding the observed association between folic acid and hemoglobin levels.

### Contributions to health, nursing or public policy

The results reinforce the importance of nutritional monitoring strategies during prenatal care, with special attention to adequate folic acid and hemoglobin levels, in accordance with national and international guidelines. Although this study did not assess the effectiveness of supplementation, the findings highlight the importance of preventive measures to reduce nutritional deficiencies during pregnancy.

## CONCLUSIONS

This study identified a low prevalence of folic acid deficiency and anemia among the pregnant women assessed. A statistically significant association was observed between hemoglobin and folic acid levels, reinforcing the importance of nutritional monitoring during pregnancy. Sociodemographic characteristics such as higher educational level, marital stability, and early initiation of prenatal care may have contributed to the observed results.

The need for new studies with more robust designs and control of confounding factors is emphasized in order to deepen the understanding of the relationship between maternal nutritional status and hemoglobin levels during pregnancy.

## Data Availability

The research data are available only upon request.
