# Supplementary material for: Correlation between folic acid and hemoglobin in pregnant women: cross-sectional study from 2017 to 2019
Source: Rev Bras Enferm. 2026 Mar 16;79:e20240568. doi: 10.1590/0034-7167-2024-0568 (PMC12991435; doi:10.1590/0034-7167-2024-0568)
Supplement: Translation

# Translation: Correlação entre ácido fólico e hemoglobina em gestantes: estudo transversal de 2017 a 2019

**DOI:** 10.1590/0034-7167-2024-0568pt

**Authors:** Tazla Ingride de Souza Lins, Geyson Alves Marinho, Vitória Caroline Santana Chaves da Silva, Myrella Maria de Sena, Suzana Lins da Silva, Malaquias Batista, Maria de Fátima Costa Caminha

**Affiliations:** I Instituto de Medicina Integral Prof. Fernando Figueira. Recife, Pernambuco, Brasil; II Faculdade Pernambucana de Saúde. Recife, Pernambuco, Brasil

**Keywords:** Anemia, Ácido Fólico, Deficiência de Ácido Fólico, Gravidez, Hemoglobina.

## Abstract

**Objetivos:**

correlacionar os níveis de ácido fólico com os de hemoglobina em gestantes.

**Métodos:**

estudo transversal, realizado entre 2017 e 2019, com 950 gestantes, baseado no banco de dados da coorte “Nutrição e infecção: o problema revisitado em função do surto de microcefalia”. Os dados foram analisados no programa Stata 12.1. Foram aplicados os testes de correlação de Spearman e qui-quadrado de Pearson, com nível de significância de 5%.

**Resultados:**

a dosagem de ácido fólico e de hemoglobina mostrou-se adequada (> 5,38 ng/ml) em 90,9% das participantes. A concentração de hemoglobina foi adequada (≥ 11 g/dL) em 80,8% das gestantes. Gestantes com hemoglobina ≤ 11 g/dL apresentaram 13,2% de ácido fólico inadequado, enquanto, no grupo com hemoglobina ≥ 11 g/dL, 8,1% apresentaram ácido fólico inadequado (p=0,031).

**Conclusões:**

observou-se associação entre os níveis de hemoglobina e ácido fólico nas gestantes avaliadas, reforçando a importância do acompanhamento nutricional durante o pré-natal.

## INTRODUÇÃO

A gestação é um período caracterizado por significativas alterações fisiológicas que requerem nutrientes essenciais para o desenvolvimento adequado do feto. Entre esses nutrientes, o ácido fólico desempenha um papel crucial, com uma demanda que pode aumentar de dez a 20 vezes em relação ao período não gravídico^(1)^.

O ácido fólico é a forma sintética do folato, descoberto pela hematologista Lucy Wills no ano de 1931 para tratamento da anemia macrocítica em mulheres grávidas^(2)^. Este nutriente é essencial para a divisão celular, crescimento dos tecidos, proliferação e crescimento de células neuronais, além da síntese de neurotransmissores^(2-4)^. O ácido fólico também é indispensável na prevenção de defeitos congênitos no tubo neural, os quais podem ser reduzidos com a suplementação desse micronutriente no início da gestação^(5)^.

A deficiência de ácido fólico ainda é um problema em muitos países ao redor do mundo, especialmente em regiões com economias de baixa renda. A fortificação de alimentos, como farinhas de trigo e milho, com ferro e ácido fólico tem sido uma estratégia eficaz para combater essa deficiência, abrangendo gestantes de menor poder econômico e gestações não planejadas^(6-11)^. Desde 1998, recomenda-se a ingestão de 400 mcg/dia de alimentos fortificados ou suplementos, além do ácido fólico obtido pela dieta^(12)^.

Além das necessidades de ácido fólico durante a gestação, são fundamentais outros nutrientes, como vitamina B12 e ferro. Suas deficiências podem causar alguns tipos de anemias^(13)^. A epidemiologia e a etiologia da anemia são multifatoriais, e envolvem uma interação complexa de causas^(14-16)^. A suplementação e a fortificação alimentar em larga escala de ferro e ácido fólico podem melhorar o estado de hemoglobina/anemia^(17-19)^.

A Organização Mundial da Saúde recomenda a suplementação de ferro e ácido fólico como medida pertencente à assistência pré-natal para reduzir perigos aos quais mãe e filho estão expostos^(20)^. Mulheres grávidas que não fazem uso de suplementos de ferro e ácido fólico apresentam um risco três vezes maior de desenvolver anemia^(21)^.

É provável que a interação com a anemia por deficiência de ferro tenha um efeito significativo de confusão na avaliação da contribuição da deficiência de ácido fólico para anemia. A deficiência de ferro causa alterações nos glóbulos vermelhos que são opostas àquelas observadas nas deficiências de ácido fólico, e a deficiência simultânea de ferro pode mascarar a deficiência de ácido fólico^(22,23)^.

Há evidências de que a concentração de hemoglobina está inversamente associada às concentrações de ácido fólico eritrocitário; no entanto, essa associação não foi encontrada em gestantes^(24)^. Dessa forma, o estudo tem como objetivo correlacionar os níveis de ácido fólico com os de hemoglobina em gestantes atendidas em hospital de referência materno-infantil para o nordeste do Brasil.

## OBJETIVOS

Analisar a correlação dos níveis de ácido fólico com os de hemoglobina durante a gestação.

## MÉTODOS

### Aspectos éticos

O estudo seguiu as diretrizes éticas estabelecidas pela Resolução nº 510/2016 do Conselho Nacional de Saúde do Brasil e foi aprovado pelo Comitê de Ética em Pesquisa com Seres Humanos do Instituto de Medicina Integral Prof. Fernando Figueira (IMIP), sob o CAAE 71259323.8.0000.5201 e número de aprovação 6.256.348, emitido em 23 de agosto de 2023. Todos os participantes forneceram consentimento livre e esclarecido por escrito antes da inclusão no estudo.

### Desenho, período e local do estudo

Trata-se de estudo transversal, com delineamento baseado em um único momento de coleta, utilizando dados de banco oriundo de coorte maior realizada entre 2017 e 2019 intitulada “Nutrição e infecção: o problema revisitado em função do surto de microcefalia”, cujo local da pesquisa foi o Centro de Atenção à Mulher do IMIP, especificamente no ambulatório de pré-natal. O manuscrito foi redigido de acordo com as recomendações do *STrengthening the Reporting of OBservational studies in Epidemiology* para estudos observacionais, garantindo a clareza e replicabilidade da metodologia utilizada.

A coleta de dados da coorte iniciou em abril de 2017, com finalização em março de 2019 pelo Grupo de Estudos Integrados da Nutrição e Saúde do IMIP. O período considerado para o presente estudo, de 2017 a 2019, corresponde ao total de 950 gestantes incluídas no banco de dados com resultados de ácido fólico e hemoglobina disponíveis.

### Amostragem e critérios de inclusão e exclusão do estudo

A amostra do estudo original foi de conveniência, sendo composta por 1.469 gestantes. As gestantes eram abordadas enquanto aguardavam a consulta de pré-natal. Os objetivos e procedimentos da pesquisa eram detalhados, e a gestante decidia sua aceitação em atender ao protocolo da pesquisa, assinava o Termo de Consentimento Livre e Esclarecido, e respondia às questões do formulário, sendo realizadas a antropometria (peso e altura) e coleta de sangue para os exames laboratoriais, incluindo o exame para ácido fólico. O resultado para o exame de hemoglobina era resgatado a partir do prontuário da gestante.

A população/amostra do estudo atual foi composta pelas gestantes do banco de dados da pesquisa original com resultado para ácido fólico e hemoglobina. Foram excluídas as gestantes que não tinham resultado para ácido fólico e hemoglobina.

Foram utilizadas variáveis sociodemográficas das gestantes (idade, raça, anos de estudo, procedência, ocupação e renda *per capita*) obstétricas (número de gestações, história de aborto, trimestre do início do pré-natal), clínicas (dosagem sérica de ácido fólico e de hemoglobina) e nutricionais (classificação de Atalah). A classificação de Atalah foi realizada com base no Índice de Massa Corporal (IMC) pré-gestacional, considerando os pontos de corte estabelecidos para cada idade gestacional, conforme o método proposto por Atalah.

A dosagem de ácido fólico foi realizada no momento da inclusão da gestante na pesquisa, independentemente do trimestre gestacional em que ela se encontrava. A dosagem de hemoglobina foi obtida a partir do resultado disponível no prontuário da gestante, sendo considerada a primeira hemoglobina registrada no período gestacional, correspondente ao momento de ingresso da gestante na pesquisa.

### Protocolo do estudo

Foi elaborado um banco *ad hoc* com as informações de interesse para responder aos objetivos do estudo atual.

### Análise dos resultados e estatística

A análise estatística foi realizada no programa Stata 12.1 utilizando o teste de correlação de Spearman e o teste qui-quadrado de Pearson. Para fins estatísticos, foi considerado o valor de p < 5%.

## RESULTADOS

A Tabela 1 descreve a frequência das características sociodemográficas das gestantes. De acordo com os dados, a maioria das gestantes tinha idade entre 20 e 35 anos, de raça parda, com escolaridade de 12 anos ou mais e residente em área urbana. Além disso, grande parte vivia em união estável ou era casada. No entanto, apesar desse perfil, mais da metade das participantes não estava inserida no mercado de trabalho no momento da coleta de dados, e aproximadamente 40% apresentavam renda *per capita* inferior a meio salário mínimo.

**Tabela 1:** Distribuição de frequência das características sociodemográficas das gestantes atendidas em hospital de referência materno-infantil para o nordeste do Brasil (abril de 2017 a julho de 2018)

Variáveis	n^*^(%)
**Idade (N = 950)**	
<= 19 anos	100 (10,5)
20 a 35 anos	708 (74,5)
36 a 47 anos	142 (14,9)
**Raça (N = 944)**	
Branca	190 (20,1)
Preta	164 (17,4)
Parda	507 (53,7)
Indígena	17 (1,8)
Amarela	66 (7,0)
**Anos de estudo (N = 949)**	
Até 8	117 (12,3)
9 a 11	117 (12,3)
12 ou mais	715 (75,3)
**Procedência (N = 950)**	
Urbano	921 (96,9)
Rural	29 (3,1)
**Situação conjugal (N = 950)**	
Solteira/separada/viúva	193 (20,3)
Casada/união estável	757 (79,7)
**Ocupação remunerada (N = 948)**	
Sim	459 (48,4)
Não	489 (51,6)
**Renda *per capita* (SM) (N = 867)**	
<1/2 SM	361 (41,6)
1/2 a 1 SM	296 (34,1)
>1 SM	210 (24,2)

SM - salário mínimo;

* A amostra variou de acordo com as informações coletadas.

A Tabela 2 apresenta as características gestacionais, antropométrica, de hábitos de vida e valores de hemoglobina e ácido fólico. A maioria das gestantes era primigesta, apresentando sobrepeso ou obesidade de acordo com a classificação de Atalah.

**Tabela 2:** Distribuição de frequência das características obstétricas, nutricionais e clínicas das gestantes atendidas em hospital de referência materno-infantil para o nordeste do Brasil (abril de 2017 a julho de 2018)

Variáveis	n^*^(%)
**Gravidez (N = 950)**	
Primigesta	367 (38,6)
Secundigesta	280 (29,5)
Multigesta	303 (31,9)
**História de aborto (N = 585)**	
Sim	243 (41,5)
Não	342 (58,5)
**Trimestre do início do pré-natal (N = 891)**	
1° trimestre	620 (69,6)
2° trimestre	271 (30,4)
**Classificação de Atalah (N = 942)**	
Baixo peso	119 (12,6)
Adequado	313 (33,2)
Sobrepeso	273 (29,0)
Obesidade	237 (25,2)
**IG na coleta de ácido fólico (N = 950)**	
1° trimestre	313 (32,9)
2° trimestre	632 (66,5)
3° trimestre	5 (0,5)
**Uso de suplemento de ácido fólico (N = 950)**	
Sim	630 (66,3)
Não	320 (33,7)
**Hemoglobina (N = 950)**	
<11 g/dl	182 (19,2)
>=11 g/dl	768 (80,8)
**Dosagem de ácido fólico (N = 950)**	
≤ 5,38 ng/ml	86 (9,1%)
> 5,38 ng/ml	864 (90,9%)

IG - idade gestacional;

* A amostra variou de acordo com as informações coletadas.

Aproximadamente 70% iniciaram o pré-natal no primeiro trimestre, indicando acesso relativamente precoce aos cuidados de saúde. Observou-se ainda que mais de 40% das participantes relataram história prévia de aborto. Quanto ao momento da coleta de ácido fólico, a maior parte das dosagens foi realizada no segundo trimestre gestacional, o que reflete a dinâmica de inclusão das participantes na pesquisa. Em relação à suplementação, cerca de dois terços das gestantes relataram o uso de ácido fólico durante a gestação (Tabela 2).

A dosagem de ácido fólico nas gestantes mostrou-se adequada (> 5,38 ng/ml) em 90,9% das participantes. A concentração de hemoglobina apresentou-se adequada (≥ 11 g/dL) em 80,8% das gestantes.

A correlação entre os valores de ácido fólico e hemoglobina apresentou o coeficiente de correlação de Spearman de 0,08, o que se traduz em uma correlação fraca.

A Tabela 3 apresenta a associação entre os níveis de hemoglobina e de ácido fólico nas gestantes. No grupo com hemoglobina ≤11 g/dL, 13,2% das gestantes apresentaram deficiência de ácido fólico. Ademais, no grupo de gestantes com hemoglobina ≥11 g/dL, apenas 8,1% delas apresentaram deficiência de ácido fólico. Esse resultado foi estatisticamente significante (valor de p = 0,031).

**Tabela 3:** Associação entre os níveis de hemoglobina e a deficiência de ácido fólico em gestantes atendidas em hospital de referência materno-infantil para o nordeste do Brasil (abril de 2017 a julho de 2018)

Hemoglobina	Deficiência de ácido fólico		Valor de *p* ^*^
	Sim	Não	
			0,031
<11 g/Dl	24 (13,2%)	158 (86,8%)	
≥11 g/Dl	62 (8,1%)	706 (91,9%)	

* *Teste qui-quadrado.*

## DISCUSSÃO

A ingestão adequada de nutrientes durante o período gestacional é de importância reconhecida pela comunidade científica^(25)^. Considerando a relevância da adequação do ácido fólico e hemoglobina para a saúde materna, o estudo atual encontrou níveis de ácido fólico e de hemoglobina adequados na maioria das gestantes.

Observou-se que a maioria das participantes apresentava características sociodemográficas que indicam maior acesso aos serviços de saúde, como escolaridade elevada e início precoce do pré-natal.

Quando comparado com a literatura, pesquisa realizada com 1.107 mulheres gestantes revelou que 42,4% apresentaram insuficiência de ácido fólico, apesar do livre acesso a suplementos de ácido fólico^(26)^. Em outro estudo, em que foram incluídas 250 gestantes, a deficiência de ácido fólico foi encontrada em 22,4%. Dessas, 4,4% utilizavam suplemento de ácido fólico, enquanto que, no presente estudo, 66,3% das gestantes relataram terem feito uso de suplemento de ácido fólico^(27)^. Outro estudo, publicado em 2023, avaliou a prevalência de anemia pelos níveis de hemoglobina em gestantes, incluindo 208 participantes, e encontrou que 42,3% tinham anemia^(21)^.

Estudos têm demonstrado que a não suplementação de ácido fólico é um importante fator associado à deficiência ou insuficiência de ácido fólico e anemia na gestação. Devido à baixa adesão de suplementos, a fortificação alimentar parece realmente importante intervenção complementar de micronutrientes^(28,29)^.

Na tentativa de avaliar os benefícios esperados da política de fortificação alimentar no Brasil, estudo transversal avaliou a prevalência de anemia na gestação antes e depois da fortificação das farinhas com ferro e ácido fólico, sendo investigadas 12.119 gestantes usuárias da rede pública de saúde em 13 municípios das cinco regiões brasileiras. Conforme a análise, não houve aumento significativo nos níveis de hemoglobina após a fortificação (p = 0,325)^(30)^.

Outro estudo recente, realizado na Irlanda, com 759 gestantes acompanhadas por meio da coorte longitudinal ROLO, avaliou a ingestão dietética de ferro, folato e vitamina B12 durante a gestação e sua correlação com os níveis de hemoglobina materna e crescimento fetal. Apesar da inadequada ingestão de ferro e folato observada em todos os trimestres, os níveis de hemoglobina permaneceram adequados nas 13ª e 28ª semanas de gestação^(31)^.

Esses dados, todavia, são conflitantes, pois estudo conduzido em Teresina, Piauí, adotou uma abordagem longitudinal para investigar a relação entre anemia e fortificação de farinhas contendo ferro e ácido fólico em gestantes. Os resultados desse estudo demonstraram uma mudança significativa nos níveis médios de hemoglobina após a implementação da fortificação. Antes da intervenção, os níveis médios de hemoglobina eram de 11,7 g/dL, aumentando substancialmente para 12,4 g/dL após a fortificação. Além disso, a prevalência de anemia diminuiu consideravelmente de 27,2%, no grupo não fortificado, para 11,5%, no grupo fortificado^(32)^.

Em outro estudo realizado em Maringá, Paraná, investigaram-se os efeitos da fortificação das farinhas na prevalência de anemia em gestantes. Foram analisados os prontuários de 366 gestantes antes e 419 gestantes após a fortificação obrigatória das farinhas. Os resultados mostraram uma baixa prevalência de anemia antes (12,3%) e após (9,4%) a fortificação, sem diferenças estatisticamente significativas. No entanto, o grupo, após a fortificação, apresentou uma concentração média de hemoglobina 0,17 g/dL maior do que o grupo sem fortificação^(19)^.

No presente estudo, observou-se que parte das gestantes não utilizou suplementação de ácido fólico durante a gestação. Não foi realizada investigação específica sobre as razões para esse fenômeno. No entanto, é possível que fatores como início tardio do pré-natal, ausência de prescrição médica e recusa da própria gestante tenham contribuído para essa situação.

Estudos sugerem que gestantes que vivem com companheiro, com maior escolaridade, primíparas e que iniciaram o pré-natal no primeiro trimestre, apresentam menor prevalência de deficiência de ácido fólico e anemia^(33-35)^, que corrobora os dados das gestantes do presente estudo e que podem ter contribuído para o resultado de baixos níveis de deficiência de ácido fólico e hemoglobina entre as gestantes. Destaca-se ainda que o estudo foi conduzido em hospital de referência, e a maioria (70,1%) das gestantes iniciou o acompanhamento de pré-natal no primeiro trimestre e, consequentemente, a suplementação do ferro e ácido fólico.

Estudo publicado em 2021, que avaliou a prevalência do consumo de suplementos de ferro e ácido fólico e fatores associados entre mulheres grávidas, incluindo 330 gestantes, encontrou que apenas 10,3% fizeram o uso correto durante o primeiro trimestre e o principal fator associado ao baixo consumo de suplementos foi o início tardio do pré-natal^(35)^.

A falta de ácido fólico prejudica a capacidade das hemácias de se manterem íntegras diante dos agentes oxidantes^(36)^. Anemias macrocíticas megaloblásticas resultam, na maioria das vezes, de deficiências de vitamina B12 e ácido fólico. Segundo estudos, antes dos programas nacionais obrigatórios de fortificação de ácido fólico e orientação de suplementação desse micronutriente no período periconcepcional, a deficiência de ácido fólico era a segunda causa mais comum de anemia durante a gravidez^(37,38)^.

Ensaio clínico randomizado demonstrou que intervenções combinadas de educação nutricional e suplementação de ferro ácido fólico trouxeram aumento de ~10 g/L na hemoglobina e redução de anemia de 69%, seguidas por queda expressiva na mortalidade neonatal^(39)^.

Outra revisão sistemática com meta-análise publicada em 2021, com um total de 439.649 mulheres nos estudos incluídos, que avaliou os efeitos da suplementação de vitaminas e minerais durante a gravidez nos resultados maternos, de nascimento, de saúde infantil e de desenvolvimento, encontrou que a suplementação de ferro junto ao ácido fólico mostrou uma redução significativa (48%) no risco de anemia materna^(40)^.

Enquanto isso, outro estudo, também publicado em 2021, avaliou se a inclusão de ácido fólico em suplementos semanais de ferro conferiu algum benefício à concentração de hemoglobina ou redução da anemia, encontrando que, após 16 semanas de inclusão de ácido fólico na suplementação, não houve diferença nas concentrações de hemoglobina^(41)^.

Vale ressaltar que, devido à ausência de fortificação com ácido fólico na Malásia, local onde foi realizado o estudo supracitado, a população de mulheres dessa região provavelmente apresenta um dos menores níveis de ácido fólico globalmente, como evidenciado pela deficiência de ácido fólico em 84% das mulheres, porcentagem muito maior em comparação ao presente estudo. Deve-se notar também que a duração do estudo foi de apenas 16 semanas. Populações com *status* basal de ácido fólico mais alto e com fortificação obrigatória podem ter uma melhor resposta nos níveis de hemoglobina^(42)^.

Em relação aos fatores que estão associados ao risco de anemia, estudo que teve como objetivo identificar características sociodemográficas relacionadas à anemia na gestação observou que as mulheres eram mais propensas a ter anemia durante o período periconcepcional quando eram mais jovens, multíparas, solteiras, com baixo nível de escolaridade, baixa renda familiar, desempregadas durante a gravidez e que residiam em área rural ou de difícil acesso a serviços de saúde^(43)^.

A baixa deficiência de ácido fólico e de hemoglobina identificada neste estudo pode ser atribuída ao fato de que a maioria das gestantes do estudo tinha mais de 19 anos, era casada ou estava em união estável e tinha 12 ou mais anos de escolaridade, residindo em áreas urbanas. Essas características sugerem uma potencial associação entre níveis educacionais mais elevados, estabilidade conjugal e uma maior probabilidade de adoção de práticas de saúde pré-natais adequadas, tais como início precoce do pré-natal e maior adesão à suplementação de micronutrientes.

Ademais, estudos demonstram que o IMC materno está associado ao risco de anemia^(44-46)^. No Brasil, gestantes com peso adequado ou com sobrepeso tiveram chances significativamente menores de serem anêmicas, em comparação com mulheres com baixo peso (adequado versus baixo peso (*Odds Ratio* 0,79; Intervalo de Confiança de 95%; 0,66 a 0,94); sobrepeso versus baixo peso (*Odds Ratio* 0,42; Intervalo de Confiança de 95%; 0,42 a 0,66). Corroborando os dados das gestantes do estudo, a maioria estava com IMC adequado (32,9%) e sobrepeso (28,6%)^(30)^.

No Brasil, a orientação da fortificação alimentar, desde a obrigatoriedade, é feita com ferro e ácido fólico^(31)^. Estudos recentes reforçam a relevância de estratégias de suplementação para redução da anemia em gestantes, incluindo intervenções baseadas em evidências para populações de baixa e média renda^(21,47)^.

A suplementação dessa vitamina e desse mineral é rotineira no início do pré-natal, podendo ter resultado na correlação significativa encontrada entre os níveis de hemoglobina e ácido fólico no presente no estudo. Por se tratar de estudo transversal, não é possível estabelecer relações de causalidade entre os níveis de ácido fólico e hemoglobina. Os achados, no entanto, reforçam a importância do monitoramento nutricional durante a gestação.

### Limitações do estudo

Este estudo apresenta algumas limitações. A principal delas refere-se à obtenção de resultados de uma amostra de mulheres que deram à luz em um hospital de referência materno-infantil, sendo que a maioria iniciou o pré-natal no primeiro trimestre.

Além disso, destaca-se que embora grande parte das participantes tenha iniciado o acompanhamento pré-natal precocemente, a dosagem de ácido fólico foi realizada majoritariamente no segundo trimestre da gestação, o que pode ter influenciado a representatividade dos níveis séricos observados. Tal característica pode ter contribuído para a baixa prevalência de deficiência de ácido fólico e os níveis adequados de hemoglobina encontrados na amostra.

Ademais, por se tratar de análise secundária de banco de dados, não foi possível incluir outras variáveis potencialmente relacionadas à deficiência de ácido fólico, como o planejamento da gravidez e o uso de suplementação no período pré-concepcional.

Por fim, ressalta-se que o presente estudo utilizou apenas análises bivariadas, sem ajuste para potenciais fatores de confusão, devido às limitações inerentes ao banco de dados disponível. Essa limitação metodológica deve ser considerada na interpretação dos resultados, especialmente quanto à associação observada entre os níveis de ácido fólico e hemoglobina.

### Contribuições para a área da saúde, enfermagem ou políticas públicas

Os resultados obtidos reforçam a relevância de estratégias de acompanhamento nutricional durante o pré-natal, com atenção especial à adequação dos níveis de ácido fólico e hemoglobina, conforme as diretrizes nacionais e internacionais. Embora o presente estudo não tenha avaliado a efetividade da suplementação, os achados ressaltam a importância de ações preventivas para redução de deficiências nutricionais na gestação.

## CONCLUSÕES

O presente estudo identificou uma baixa prevalência de deficiência de ácido fólico e de anemia entre as gestantes avaliadas. Observou-se uma associação estatisticamente significativa entre os níveis de hemoglobina e ácido fólico, o que reforça a importância do monitoramento nutricional durante a gestação. Características sociodemográficas como maior nível educacional, estabilidade conjugal e início precoce do pré-natal podem ter contribuído para os resultados observados.

Ressalta-se a necessidade de novos estudos com delineamentos mais robustos e controle de fatores de confusão, a fim de aprofundar a compreensão sobre a relação entre o estado nutricional materno e os níveis de hemoglobina durante a gestação.

## DISPONIBILIDADE DE DADOS E MATERIAL

Os dados de pesquisa só estão disponíveis mediante solicitação.
